# “Condoms are … like public transit. It’s something you want everyone else to take”: Perceptions and use of condoms among HIV negative gay men in Vancouver, Canada in the era of biomedical and seroadaptive prevention

**DOI:** 10.1186/s12889-019-6452-7

**Published:** 2019-01-28

**Authors:** Benjamin J. Klassen, Karyn Fulcher, Sarah A. Chown, Heather L. Armstrong, Robert S. Hogg, David M. Moore, Eric A. Roth, Nathan J. Lachowsky

**Affiliations:** 1 Epidemiology and Population Health, British Columbia Centre for Excellence in HIV/AIDS, Vancouver, BC Canada; 2 Department of History, Simon Fraser University, Burnaby, BC Canada; 3 School of Public Health & Social Policy, University of Victoria, room B202, HSD Building, 3800 Finnerty Rd, Victoria, BC V8P 5C2 Canada; 4 YouthCO HIV & Hep C Society, Vancouver, BC Canada; 5 Faculty of Health Sciences, Simon Fraser University, Burnaby, BC Canada; 6 Faculty of Medicine, University of British Columbia, Vancouver, BC Canada; 7 Department of Anthropology, University of Victoria, Victoria, BC Canada; 8 Centre for Addictions Research of British Columbia, Victoria, BC Canada

**Keywords:** Condoms, Seroadaptive strategies, HAART, Gay and bisexual men, Combination prevention, Social ecological model, HIV prevention

## Abstract

**Background:**

The emergence of biomedical and seroadaptive HIV prevention strategies has coincided with a decline in condom use among gay men.

**Methods:**

We undertook a social ecological analysis of condom use and perceptions using nineteen semi-structured interviews with HIV negative gay men in Vancouver, Canada who used HAART-based prevention strategies.

**Results:**

Contributors to inconsistent condom use were found at various levels of the social ecological model. Ongoing concern regarding HIV transmission and belief in the proven efficacy of condoms motivated contextual use. When condoms were not used, participants utilized seroadaptive and biomedical prevention strategies to mitigate risk.

**Conclusions:**

These findings indicate that notions of “safety” and “risk” based on consistent condom use are eroding as other modes of prevention gain visibility. Community-based and public health interventions will need to shift prevention messaging from advocacy for universal condom use toward combination prevention in order to meet gay men’s current prevention needs. Interventions should advance gay men’s communication and self-advocacy skills in order to optimize these strategies.

**Electronic supplementary material:**

The online version of this article (10.1186/s12889-019-6452-7) contains supplementary material, which is available to authorized users.

## Background

Despite concerted prevention efforts and the increased availability and uptake of biomedical advances, gay and other men who have sex with men (henceforth gay men) are disproportionately affected by the Canadian HIV epidemic, representing approximately half of all new diagnoses nationally [1, 2]. In British Columbia (BC), rates of new diagnoses among gay men have remained consistent despite a decline in overall numbers of new diagnoses [3], with consistent increases in diagnoses among young gay men (born after 1980) of particular concern [3, 4]. A 2016 study of gay men in Vancouver, BC estimated that 23.4% were HIV positive, of which an estimated 18.6% had an unsuppressed viral load [5]. Until recently condoms were synonymous with HIV prevention among gay men, but condom use is now declining and condomless anal intercourse (CAI) is on the rise both in total incidences and the number of different CAI partners over time [6–10].

Scholars have posited a number of explanations for this decline in condom use. Some have suggested that three decades of condom-based prevention messaging and interventions have caused safer sex fatigue or burnout, leading to inconsistent condom use. New interventions that focus solely on condoms may be viewed as ineffective or unrealistic, and may therefore be rejected, particularly among older gay men who are tired of prevention messaging focused primarily or exclusively on condoms [11–15].

The growth of a “barebacking” (intentional CAI) subculture is another potential contributor to inconsistent condom use. Studies have noted the growing prevalence of “bareback”-identifying gay men who are aware of the risks of CAI but choose to have condomless sex regardless, possibly due to the payoff of increased sexual pleasure [16–18], though motivations vary by age and ethnicity [19]. As “barebacking” has become normalized, with help from media like pornography [20], non-“barebacking” gay men may feel pressure to engage in CAI [17, 21].

Emotional and erotic factors may also influence condom use practices. Condoms are frequently perceived as a barrier to sexual pleasure and arousal, whereas CAI is viewed as facilitating sensation, physical pleasure, and deeper levels of eroticism and lust [21–24]. A desire for trust, intimacy, commitment, and love may provide further motivation, wherein CAI serves as a manifestation of these emotions and a representation of the depth of a given partnership [21, 23, 25, 26]; condoms by contrast are seen as a barrier to intimacy [7].

Existing literature also highlights the impact of substance use on consistent condom use. Excessive consumption of alcohol and crystal methamphetamine use in particular have been shown to increase incidences of CAI, with binge drinking and club drug use potentially leading to particularly high-risk encounters with casual, HIV-serodiscordant partners [27–29].

The final and perhaps most significant factor influencing condom use is the emergence of highly active antiretroviral therapy (HAART) - based biomedical interventions: Treatment as Prevention (TasP) – using HAART to treat HIV positive individuals and lower individual and population-level viral loads [30]; Pre-Exposure Prophylaxis (PrEP) – consistent use of HAART by HIV negative individuals to prevent seroconversion in case of exposure [31]; and Post-Exposure Prophylaxis (PEP) – a 28-day course of HAART taken by HIV negative individuals immediately following potential exposure to prevent seroconversion [32]. One explanation for the influence of HAART on condom use is a growing sense of “optimism” as HIV has been transformed from a death sentence to a chronic condition [11, 33]. This optimism may be due to the perception that HAART has made HIV more manageable and less frightening, or a result of a perceived decrease in the likelihood of transmitting or contracting the virus [33]. As antiretrovirals have been proven to be an effective prevention tool, incidences of CAI among gay men have increased, indicating that optimism may shift the relative balance of risk and pleasure in gay men’s sexual decision-making [6, 11, 33, 34].

Other scholars have explained the relationship between declining condom use and availability of biomedical interventions using the concepts of behavioural disinhibition – which emphasizes the emotional or physical determinants of sexual behaviour – and risk compensation – which emphasizes the rational weighing of risk in sexual decision-making [22]. Biomedical advances have potentially shifted both the rational risk balance and existing self-imposed behavioural constraints to the extent that the corresponding increase in CAI may offset the potential benefits of HAART-based interventions [22, 35]. The evidence for this explanation is conflicting, however; although condom use has increased among PrEP clinical trial participants, [36, 37] evidence for risk compensation outside the clinical setting is inconclusive [37, 38]. Some non-clinical studies have documented decreases in condom use ranging from 23% to 60% [15, 35, 39–41]; in other analyses, participants did not believe that access to PrEP would affect their own condom use, but believed that other gay men would likely have more CAI [8, 42]. A study of highly sexually active gay men in New York found little evidence of risk compensation, with the exception of a small, particularly at-risk group of gay men who were already having CAI, making them prime PrEP candidates [15]. Studies of PEP use have similarly found little support for risk compensation, with many participants describing their experience with PEP as a “wake-up call” that ultimately led to more consistent condom use [9, 43].

The development of various seroadaptive prevention strategies that enable CAI while mitigating sexual risk may be further affecting rates of condom use [44]. These strategies are possible because of biomedical advances, and include: serosorting – seeking seroconcordant partners or selectively engaging in specific sexual behaviours based on a partner’s known HIV serostatus [7]; viral load sorting – actively seeking partners with low or undetectable HIV viral load or using this information to determine the type of sexual beahviours between serodiscordant partners [33, 45]; negotiated safety – monogamy agreements between primary partners or the exclusion of high-risk behaviours outside of the primary partnership [19, 46]; and strategic positioning – taking the insertive role as an HIV negative partner in CAI with HIV positive or unknown status partners [44]. While these measures are increasingly common among gay men, their effectiveness is not well-studied; furthermore, these strategies rely on explicit communication, disclosure, and trust, none of which are guaranteed in a given sexual encounter [19]. Rather than communication and trust, many gay men tend to assume or infer the HIV status of their partners without explicit conversation [21]. Further, negotiated safety agreements are not always upheld as a large proportion of HIV transmissions (as much as 68%) occur within the context of primary relationships [47–49].

In this paper, we offer a further qualitative contribution to the literature on condom use trends and perceptions with a particular focus on the impacts of HAART-based interventions on gay men’s HIV prevention practices. Using a social ecological model of health [50], we examine the current place of condoms within gay men’s sexual risk management practices, and explore the motivations for their use and disuse. We then suggest possible improvements to current sexual health interventions that take into account the various interrelated factors affecting condom use among gay men and the shifting definitions of sexual safety and risk.

## Methods

Participants were drawn from the Momentum Health Study, a longitudinal bio-behavioural prospective cohort study of HIV positive and HIV negative gay men in Metro Vancouver, BC, Canada (inclusion criteria: age ≥ 16 years, live in Metro Vancouver, identify as a man (including trans men), have had sex with a man in the last six months, and able to answer a questionnaire in English) [5, 51]. Participants were eligible to take part in an interview if they were HIV negative and had reported prior use of PEP, PrEP, and/or viral load sorting. In total, nineteen individuals ranging from the ages of 22 to 58 (median average: 36 years) agreed to be interviewed. Trained peer research associates [52] who had prior experience working with gay men conducted the interviews. Participants provided written informed consent prior to the interview, and an honourarium of $50 CAD was given upon interview completion.

Interviews were semi-structured [53]; questions focused on participants’ perceptions and use of various HIV prevention strategies, particularly PEP, PrEP, and viral load sorting [see [54]. Participants were asked to describe what HIV prevention meant to them, what strategies they utilized to prevent HIV, and how they would compare the overall effectiveness of various prevention strategies. Finally, they were asked to consider how their knowledge of biomedical prevention strategies had affected their conceptions of sexual “risk” and “safety,” particularly with respect to condom use.

Interviews were audio recorded, transcribed verbatim, anonymized, and coded through in-depth thematic analysis Additional file 1. To maximize the trustworthiness of our analysis, we followed Lincoln and Guba's evaluative framework for qualitative work via the establishment of credibility, transferability, dependability, and confirmability [55]. Four readers generated a master code list based on multiple readings of the transcripts, with frequent discussion to ensure consistency. At least two readers coded each interview and discrepancies were resolved within the dyad or via a third coder. Codes were revised and expanded as needed through an iterative engagement with the data [54] and grouped into themes and subthemes. We then organized these themes within a social ecological model based on McLeroy and colleagues’ [50] outline of five stratified factors impacting health promotion; while McLeroy and colleagues distinguish between institutional and community factors, we chose to merge the two. On the interpersonal level, we focused on interactions and relationships with sexual partners, as these were most frequently cited by our participants as influencing their condom use practices.

## Results

Of the nineteen HIV negative participants, eight had used PEP, seven had used TasP-facilitated viral load sorting, and two were using PrEP at the time of the interview. Each of the quotes presented below are identified by a pseudonym and the participant’s reported age.

Overall, participants expressed a range of complex and often conflicting perspectives on condoms, from outright disdain to approval. However, a general rejection of consistent condom use was evident, and contextual condom use within a combination prevention framework was the norm. First, we examine participants’ perceptions and use of condoms, beginning with the intrapersonal or individual factors they identified as contributing to inconsistent use and building to the policy-level. We then outline participants’ motivations for continued condom use within a combination HIV prevention framework.

### Social Ecological Factors in Perceptions and Use of Condoms

#### Intrapersonal (Individual) Factors

McLeroy and colleagues [56 p355] describe intrapersonal factors as “characteristics of the individual such as knowledge, attitudes, behaviour, self-concept, skills, etc.” Participants in this study described a number of these individual factors as influencing their condom use, including the desire for increased sexual pleasure, eroticism and freedom, substance use, and perception of risk.

For many participants, condoms inhibited physical pleasure and they therefore disliked using them. For Curtis (age 25) sex without condoms simply felt better: “it’s hard to get used to … wear [ing] condoms when … the physical pleasure is so much more with not having a condom.” In addition to decreasing physical sensation, condoms were viewed as impeding sexual performance, as Jordan (age 25) noted: “a lot of guys when they hear the word ‘condom’ or just hear that wrapper, they immediately lose their hard-on there.” This suggests that condoms were also perceived not only as a physical challenge but as a challenge within the emotional realm of eroticism and sexual desire. CAI was deemed more exciting, with some participants, including Malcolm (age 34), noting that riskiness could itself be perceived as erotic: “there was probably an element of risk involved which, unfortunately, is quite exciting … [CAI is] more enjoyable.”

Ideas about sexual freedom and liberation were additional factors in inconsistent condom use. Some participants viewed condoms as a barrier to sexual liberation and expression, and highlighted their desire to explore sex more fully through CAI. Aaron (age 46) wanted to explore the “deeper and darker … parts of sexuality” and “push … the limits of the traditional ideas of sex” through HAART-mediated CAI. In addition to the “pull” of the liberating potential of CAI, participants also experienced a “push” away from condoms due to their association with anxiety and fear around sex, as Bob (age 58) indicated:

anything that … reduces the anxiety around sex and allows it to be this wonderful expression and experience of life is a really positive force … if the treatments are allowing that, permitting that … with the absence of anxiety and stress around it, the world is going to be a better place …

For Bob, condoms represented anxieties around sex whereas HAART-facilitated CAI allowed for a fuller, more positive expression of sexuality. Given this contrast, participants’ rejection of consistent condom use is not surprising. Indeed, many participants expressed fatigue and frustration with condom use, especially those who had been practicing safer sex for many years. While they acknowledged that condoms were effective, several participants described sex with condoms as “boring” (Aaron, age 46) or “unsatisfying” (Bryan, age 49). Dan (age 46) explained, “I’ve always understood the necessity for condoms but I’ve always hated them; they take so much away from the experience and make it such a pain that in a lot of cases they just made it not worth it.”

With regards to individual behaviour, substance use strongly impacted condom use and sexual decision-making. Many participants stated that when using substances, they took sexual risks with which they would otherwise not be comfortable. As Russell (age 26) described: “I was into a lot of drinking and drugs and I was just really being very unsafe in general … becoming conscious to that situation really had me concerned.” In Kevin’s (age 31) opinion, “the drugs or the desire for pleasure exceeds the precaution in that moment. And I’ve regretted those decisions, very much so.” By altering the relative value of risk and pleasure in the decision-making process, participants felt that substance use decreased the likelihood of condom use in a given sexual encounter.

The final intrapersonal factor impacting condom use for participants in this study was their perception of HIV risk. Several expressed what Prestage and colleagues [33] refer to as treatment optimism, stating they were “less fearful” (Will, age 28) due to treatment advances that had rendered HIV a chronic condition, the management of which one participant likened to “brushing your teeth before you go to bed” (Bryan, age 49). For Kevin (age 31), HIV was

… no longer a death sentence, so even though the stress of becoming positive is still there, the severity of it, the weight of it, is no longer as strong or as heavy as it once was because there are solutions … I have a lot of friends who aren’t detectable … and they’re leading good lives.

The transformation of HIV into a treatable, livable condition deeply impacted participants’ concerns over HIV transmission and influenced their condom use habits as a result.

Other participants demonstrated transmission optimism [33], explaining that the availability and perceived efficacy of HAART-based interventions contributed to their views that consistent condom use was unnecessary, due to perceptions that the chances of HIV transmission were low. Curtis (age 25) was unconcerned about sex with partners who were currently using HAART because of his belief in the effectiveness of these treatments: “if … they’re on PrEP or they’re … undetectable … I don’t really think about it … I don’t really see that there will be a … transmission, therefore there’s nothing to prevent.” Notably, this “nothing to prevent” seemed to extend to other STIs, against which biomedical HIV preventions offer no protection, although participants did not typically express any concern over this.

#### Interpersonal (Relationship) Factors

On the interpersonal level, participants’ perceptions of condoms were primarily shaped by their interactions with sexual partners. In encounters with new partners, several participants emphasized the need to maintain the mood or the “heat of the moment”, noting that discussing sexual safety and potential condom use disrupted or inhibited sexual interactions. Bob (age 58) felt that asking about HIV status “breaks the whole mood of sex and … how far you're going to go and what you're going to do … just by asking the question, it kind of for me resulted in holding back.” The “heat of the moment” could also result in a decrease in the perceived importance of safety, which was the case for Will (age 28): “I'm conscious of having safe sex, and that's important to me, and then I find myself in the moment it's not important to me, and … I like the way it feels without a condom, so I push past that.” For the participants in this study, “good sex” and explicit conversations regarding condom use were often incompatible.

A small number of younger participants mentioned that imbalances in relationship power dynamics and the ceding of sexual decision-making to partners could contribute to reduced condom use. These young men indicated that pressure or coercion from sexual partners could lead to CAI, especially when sex was negotiated on uneven terms. Russell (age 26) noted:

If somebody is a little more submissive, then I know there are some people that kind of try to bully people into not using protection or peer pressure or if there’s somebody they really feel a connection to and then say, yeah, but no condoms, period.

The suggestion that peer pressure and the threat of withholding sex could motivate CAI – especially when the partner advocating for condom use lacked sexual agency – described Kevin’s (age 31) experiences as well:

[It’s] like a back and forth, because I would like to use them [condoms]; well, I don’t want to use them. Well, I would like to, you know, can you try not using? No … And for the most part I end up not using them, because I feel like kind of … not pressured, but I want to have the sex.

In these instances, Kevin felt that his desire to have sex, even on terms with which he was not entirely comfortable, outweighed the risks of CAI and he therefore agreed to forego condom use despite his initial reservations.

Within primary relationships or for participants in search of such relationships, the pursuit of emotional intimacy was frequently cited as a reason for abandoning condom use due to the perception that CAI facilitated “closeness” (Kevin, age 31) and “more natural” sex (Curtis, age 25). According to Chris (age 51) this desire for intimacy could be a stronger motivator than physical pleasure:

… it's almost to the point [where] there's really no point without it being raw, without it being bareback … let's face it, it's not a huge feeling difference … But it's a huge difference for me in my mind, like the intimacy factor …

Related to the desire for intimacy and closeness, participants argued that CAI could provide an opportunity to build trust with a partner, since condomless encounters required a greater degree of communication. Curtis (age 25) explained:

[condomless encounters are] probably going to be much more of an opportunity to learn about things and maybe almost gets you closer with other people because it’s all … a trust thing, right? … I trust them and they can see that I trust them … it just kind of builds trust in a weird way. I feel like having sex with people definitely builds trust, always.

Here, Curtis describes trust not just as a motivation or requirement for CAI, but as something that could be expressed and developed through CAI. Within the context of longer-term relationships, not only did trust and intimacy facilitate CAI, but CAI could serve an important emotional function in interactions with partners.

One strategy that participants described as an alternative to practicing consistent condom use within primary partnerships was negotiated safety agreements that either ensured monogamy or placed limits on risky sexual behaviour outside of the relationship that could lead to seroconversion. In some cases, these agreements entailed getting tested and agreeing to monogamy with a partner before ceasing to use condoms, as Jordan (age 25) did: “I am only with one partner … when we decided that we wanted to be in a monogamous relationship [we agreed] to go … and get tested together and be able to drop the condom.” Monogamy was viewed as an ideal means of safely engaging in CAI by a few participants, although they acknowledged the potential for dishonesty. Bryan (age 49) stated

… when I’m with a partner … basically I flat out tell them that I want to be in a monogamous relationship. I don’t want to be sleeping with other people. But if I did ever sleep with somebody else I would tell you before I had sex with you again just out of pure respect for your sexual health and my sexual health … And I would want them to do the same thing to me.

For other participants, these agreements could involve CAI with more than one main partner while having the option of casual anal sex using condoms outside of these partnerships, as was the case for Dan (age 46): “If I am ever again going to play with somebody … other than my husband or boyfriend, I will use a condom with them. I’m just not willing to take that risk.” In addition to testing and monogamy, participants managed risk with casual partners through a variety of measures other than condom use, as George (age 51) explained: “we try to look for people that are negative or undetectable and we tend to just be tops only. But do whatever with each other.” Regardless of the details of their safety agreements, participants viewed such agreements as safely facilitating CAI within primary partnerships.

#### Social (Community) Factors

Adding to the intra- and interpersonal factors affecting participants’ views on condoms were community-level factors, such as shifting norms around condom use, “barebacking,” and seroadaptive strategies. Dislike for condoms was not only an individual preference; participants suggested that their personal views reflected shifting community norms, though the extent to which this shift was in fact occurring or was overemphasized to legitimate personal desires for CAI is difficult to ascertain. In contrast to this personal preference for CAI, however, participants often expected that others would continue to use condoms, as Scott (age 48) stated:

Condoms are one of those things that, you know, like public transit. It’s something you want everyone else to take … They don’t like using them themselves necessarily but they certainly don’t think anybody else should be able to get away with not using them.

As well as highlighting the difference between men’s personal choices around personal condom use and their expectations of others’ use, Scott’s statement provides another example of the contrast between many gay men’s beliefs or intentions around condom use and their actual behaviours. The gap between behaviour and intention led to some participants questioning how effective condom-based strategies could be at a community level when used inconsistently. As Wayne (age 36) observed, “condoms … also depend on people being adherent and we know that regular condom use has been on decline for many years in the gay community.”

Participants’ condom use and perceptions were additionally influenced by the increasing prevalence of barebacking. Bob (age 58) remarked that this shift had occurred in a relatively short period of time: “ … certainly five years ago you would just never come across it. And today … the number of requests to bareback is just … exponentially over where we were two years ago.” In Scott’s (age 48) experience this shift had been so dramatic that “I really don’t know anybody who uses condoms. Like, I can hardly think of anybody.” Summarizing the impact of community norms on individual behaviour, Wayne (age 36) noted that the “social acceptability of barebacking seems to have evolved.”

The increasing prevalence of seroadaptive strategies within the gay community was mentioned by several participants as further evidence of shifting community norms. Along with negotiated safety agreements and viral load sorting, serosorting and strategic positioning were used, offering viable alternatives to condoms. For some, these strategies were presented as methods that could virtually eliminate risk. Cody (age 22) felt that this was the case for serosorting: “sex between people who are communicating their statuses isn’t risky at all because you know what's going on.” Similarly, George (age 51) expressed his confidence in the efficacy of strategic positioning for himself and his primary partner: “because we're basically only topping, I'd say we actually probably don’t care at all [about our partners’ HIV statuses].” Whether this level of confidence reflects community norms and/or an attempt to legitimize personal preferences, the frequency with which participants referred to these strategies indicates that they had gained a great deal of traction within the community as an alternative to condoms. While several participants questioned the efficacy of serosorting, most agreed that strategic positioning was effective, including Wayne (age 36), who noted “I mostly top … I think it's reasonably well-established that that is generally a lower risk than bottoming.” The description of strategic positioning as an “established” means of risk reduction supports the notion that seroadaptive strategies have become a norm within the gay community.

Within the broader context of shifting community norms, participants noted that specific social situations – such as the bathhouse setting – could contribute to decreased condom use. In their view, the bathhouse was a context in which CAI was expected, legitimizing personal preferences for CAI. Bob (age 58) cited an experience of receptive condomless group sex in a bathhouse as an example of how environment factored into the choice to engage in CAI: “Sometimes in sex you just can be totally taken away … so in the bathhouse, for example, I can just totally lose myself in sex … I wasn't expecting it, it just happened out of the blue, but I didn't stop it, that's the important thing.”

#### Policy Factors

On the policy level of social ecological factors in health [50], participants’ attitudes towards condoms were shaped by the availability and quality of HIV treatment in BC, as well as their knowledge of these treatments, which was largely facilitated by various innovative community-based organizations [56]. Since 2010, TasP has been actively promoted in BC, with free HAART available to HIV positive individuals province-wide [51]. This policy clearly played a role in participants’ overall optimism regarding HIV, which they generally viewed as a treatable chronic condition due to the accessibility of HAART. While most did not explicitly state that public policy had contributed to their personal optimism, some did directly acknowledge the impact of policy on community and personal perspectives. Aaron (age 46) explained, “the treatment for maintaining … undetectab [ility] is of such high quality here in BC, and if your story is about you know just how great the care is … maybe the idea of turning positive isn't as scary as it once was.” For Aaron and many other participants, the proven efficacy and wide availability of HAART in BC had transformed seroconversion from a death sentence to a treatable condition, rendering HIV much less frightening than it had been in the past. Aaron went on to explain that

[HAART-based] strategies and these options that are out there now have definitely redefined

the traditional definitions of safe sex and riskier sex … I just think that people are … definitely redefining what they consider to be risky or what was considered risky has now become [safer].

Based on Aaron’s statement, policies that have increased the availability of TasP, PEP and PrEP have shifted both community- and individual-level perceptions of risk and safety surrounding HIV transmission, fundamentally altering the meaning of risk and safety. Aaron’s remark clearly demonstrates how policy-level factors interact with intrapersonal, interpersonal, and community factors to produce observable impacts on health behaviours.

In addition to reducing the fear of HIV transmission, policy shaped participants’ perceptions of the relative risk posed by HIV positive undetectable partners. While not all participants agreed on the efficacy of serosorting as a prevention strategy, viral load sorting – seeking out HIV positive undetectable partners – was deemed an effective means of prevention and an excellent alternative to condom use by many. Most participants expressed a preference for undetectable partners over HIV negative partners, and displayed considerable knowledge about TasP and undetectability that reflected the unusually high rates of TasP awareness among gay men in Vancouver generally [51]. Chris (age 51), noted his confidence in viral load sorting as a prevention strategy, although he believed that viral load measurements did not always accurately reflect infectivity: “The transmission for that is extremely low as well. Even though it’s … done with things like saliva and blood whereas … [I'm more interested in] semen because the concentration of that I'm thinking would probably be a lot higher.”

Among participants who preferred viral load sorting to serosorting, the reasoning for this preference varied. One reason was the view that undetectable individuals were more likely to be aware of their sexual health in general, as Curtis (age 25) explained:

… they have to get tested every month, every three months or something. They do routine blood work, which means they’re probably going to catch any other STI’s they have, therefore … they’re most likely going to be … either STI free … or they’ll be treated.

This statement points to the possibility that the availability of TasP in BC has contributed to the perception that not only are HIV positive undetectable individuals “low risk” with regards to HIV transmission, but lower risk in terms of all STIs. On a related note, Aaron (age 46) felt that the current, detailed sexual health information undetectable partners could provide as a result of routine testing made them more trustworthy than those who claimed to be HIV negative:

when it comes to negative guys … it can be a little bit harder to take their word for it. But … when somebody is sharing their status and their viral load and all those things with me, I feel there’s a level of honesty there … I could be giving too much trust, possibly … but I feel safer in that situation than with negative guys.

Overall, provincial public policy that has made TasP widely accessible and highly visible was an important factor in participants’ adoption of viral load sorting as a means of HIV prevention, demonstrating the considerable effects of policy decisions on individual perceptions and behaviour.

Provincial public policy also had a substantial impact on the uptake of PrEP and PEP among participants, albeit in a less positive manner. In contrast to the active promotion of TasP, participants noted that barriers to PEP and PrEP access and promotion were prominent at the policy level. For example, cost could hinder access to PrEP, and for both PrEP and PEP, interactions with healthcare providers, some of whom may be reluctant to prescribe these interventions, created an additional barrier. Regarding PEP, Scott (age 48) noted:

[PEP] doesn’t seem particularly effective … you have to go to Emergency, wait around for hours and hours … And then be shut down half the time … if it was accessible as hell we could just walk to the pharmacists and say give it to me … But that’s not how they do it.

Scott’s description of the arduous process of securing PEP contrasted sharply with participants’ general portrayal of TasP promotion, revealing gaps that may need to be addressed at the policy level.

### Contextual Condom Use and Combination Prevention

Participants held complex and conflicting perspectives on condom use, shaped by numerous factors at all levels of the social ecological model of health, and most participants continued to use condoms in certain situations. While they generally felt that prevention strategies other than condoms could be highly effective, many nevertheless expressed continuing anxiety regarding HIV transmission. Jamie (age 30) noted that “I was very anxious about making sure that I don’t get HIV and like if there was any possible chance, even like 0.1%, I don’t want it.” This anxiety was particularly evident when participants described sexual encounters in which they had chosen not to use condoms, acknowledging the stigma that still existed around HIV seroconversion, and referring to condoms as a proven method of risk reduction that offered “peace of mind”. Although they may have had confidence in other methods, when asked to compare the effectiveness of various options the majority of participants agreed that condoms were either very effective or the most effective form of prevention.

For some participants, the accessibility of condoms in comparison to the relative inaccessibility of biomedical interventions factored into their perceived effectiveness. The issue of accessibility was compounded in some participants’ views by worries that the growing prevalence of biomedical interventions would have adverse effects on prevention efforts as a whole. Cody (age 22) was concerned that new interventions might lead to decreased communication between partners: “PrEP makes sex much less risky but also is something that makes you not have to communicate as much either.” Other study participants felt that the potential lack of communication could increase levels of sexual risk taking. Bob (age 58) suggested that the increase in CAI facilitated by PrEP would potentially lead to a net increase in HIV transmission: “HIV is still real, it is still being transmitted presumably … allowing for [greater] access to sex suggests to me that there is a possibility of … higher transmission rates.”

In contrast to the perception of condoms as a proven prevention method, participants discussed biomedical treatments in more conflicted terms. They had similar concerns regarding seroadaptive strategies, predominately due to the reliance on accurate, trustworthy disclosure. Several participants noted that casual partners may be dishonest about their serostatus or may be unaware of their status, therefore undermining serosorting as a prevention strategy. As Curtis (age 25) put it, “no one is really HIV negative unless you got tested that second and had sex the next minute, right? Because that’s the only way of knowing you had sex with someone else who is HIV negative.” Participants had similar worries regarding communication with undetectable partners: Scott (age 48) suggested, “You have to know a person very, very well to have much confidence in [treatment as prevention].” This statement indicates that relying on TasP disproportionately placed the burden of responsibility on the HIV positive partner; while this was seen as a benefit by some participants, it produced anxiety in others.

Participant perspectives on biomedical prevention strategies as an alternative to condom use further varied depending on the specific method in question. Among PEP-experienced participants, condom use was reinforced by the experience of seeking out biomedical treatment. This group described PEP as a “wake-up call” that highlighted the potential consequences of CAI and the value of consistent condom use. Using PEP offered a window into the burdens of HAART-based treatment and the necessity of adherence for HIV positive individuals, as Aaron (age 46) acknowledged:

even having to do the regimen of PEP, I think there was three different pills you had to take and it really … made me aware of wow … this could be a lifelong part of your life, right? Not that was good or bad but it just was something that would impact your original previous way of living.

Participants also noted the anxiety and fear of these experiences and wished to avoid those feelings in the future. Kevin (age 31) stated, “If I were to choose to have sex … in a casual setting, I would have a condom, because I don’t want to go through that stuff again, the stress of not knowing whether I’ve contracted something.” Cameron’s (age 26) experience with PEP likewise reaffirmed his decision to use condoms: “you just kind of look out for yourself more, or you just don't make stupid decisions … I mean you know you never want to repeat that ever again, so, yeah it is more of a conscious choice.”

Despite their concerns about the effectiveness of biomedical and seroadaptive strategies, many participants used condoms only in casual, higher-risk encounters while relying on other forms of prevention in other contexts. Russell (age 26) noted:

I think the best method, in terms of random hook-ups or bath houses, parties, things like that; I think, really, condoms is the best way to go just because it’s accessible. Anybody can use it, really. It’s just really practical … if we’re going to hook-up just because we’ve had a few drinks or the chemistry is right or feeling casual that day or whatever … condoms is typically my go-to.

Russell’s statement suggests that the accessibility of condoms and their utility within casual encounters may account for their continued use in certain contexts. Participants also acknowledged that no singular form of prevention was completely effective and suggested that combining multiple strategies provided the most protection. For some, including Wayne (age 36), the combination of PrEP and condoms offered “reassurance”:

PrEP is not 100 percent certain either … but it's a great deal more certain than most of the other options … there are some who say it's more certain than condom use, if you assume adherence is high on PrEP side and if you accept that adherence on the condom use side is - and correct use of condoms and breakage and all those sorts of things - is not 100 percent. So it - it certainly has added a significant layer of reassurance.

Other participants combined forms of prevention as an alternative to condoms. In Dan’s (age 46) case, he and his HIV positive partner employed a combination of strategies that he felt rendered condoms unnecessary:

My boyfriend is positive, but he’s undetectable and he and I both get checked every three months, just in case, and I always top, and I feel comfortable with that; I’m not worried about catching anything from him with that kind of a setup.

By combining viral load sorting, strategic positioning, and frequent testing, Dan and his partner were able to manage risk without condoms, despite being in a relationship that may have typically been considered “high-risk.” Presumably, this enabled Dan to optimize pleasure and intimacy within his relationship while still allowing for “comfort” in terms of sexual safety and the minimization of risk.

## Discussion

Consistent with previous research, participants in this study cited numerous social ecological factors that motivated CAI and inconsistent condom use: the desire for intimacy and pleasure, condom fatigue, the normalization of barebacking, feeling unable to negotiate with partners, event-level loss of control due to drug use or “heat of the moment” sexual urges, and the belief that biomedical interventions offered an effective alternative to consistent condom use. When not using condoms, participants employed various seroadaptive strategies to reduce risk, including negotiated safety agreements, serosorting, viral load sorting, and strategic positioning. While biomedical interventions and seroadaptive strategies could facilitate CAI and sexual exploration, many participants expressed anxiety over the efficacy of these strategies in light of ongoing HIV transmission concerns. As a result, despite generally disliking condoms, most participants acknowledged that they were a highly effective form of prevention and continued to utilize them contextually, particularly with higher-risk, casual partners.

With regards to biomedical prevention strategies, HAART did influence participants’ condom use, but the nature of this impact varied based on the form of treatment participants accessed. PrEP and – to a lesser extent – TasP were associated with substantial decreases in consistent condom use, and participants felt on the whole that these strategies were useful alternatives to condoms. These results support previous studies that associate PrEP use outside of clinical trials with risk compensation and declining condom use [15, 35, 39–41]. Within clinical PrEP trials, where PrEP access is paired with extensive risk counselling, this decline in condom use has not occurred [36, 37], indicating the potential value of packaging broader PrEP rollout with extensive HIV prevention education and behavioural interventions [8, 22, 35, 38, 40]. In contrast to the risk compensation found among PrEP and TasP users, risk compensation has not been found among PEP users [9, 43], and this was true of participants in this study who had used PEP but not PrEP or TasP. The condom-affirming, “wakeup call” aspects of PEP may be due to its perceived role as a “last resort” to be used only when other forms of prevention fail or during abnormal lapses in prevention regimens [43]. Additionally, the relative intensity and associated side effects of HAART dosing under a PEP regimen may serve to discourage individuals from having to use PEP again, reaffirming their commitment to consistent condom use [43].

The findings of this study also align with recent research that highlights gay men’s balancing of risk and pleasure within the context of an expanding arsenal of prevention tools, and the influence of numerous socioecological factors on individual behaviour. Contradicting the assumption of HIV optimism and risk compensation models, however, declining rates of condom use may not be due to declining concerns about HIV risk but may instead be a result of having a wider variety of prevention methods to draw from in ensuring sexual safety. The majority of participants in this study expressed considerable concern about HIV transmission, even as they described it as a chronic, treatable condition, a finding consistent with existing scholarship [12, 57]. While gay men may still be concerned about HIV transmission, this expansion of prevention options has broadened the concept of sexual “safety” [23]. As a result, interventions that focus on traditional definitions of safer sex and condom use do not reflect the breadth of prevention strategies now utilized by gay men, nor the influence of social environments on safer sex practices, and may therefore be less likely to be used by many. Given the imperfect protection offered by condoms [58] and the number of prevention options available, HIV intervention work should strive to advance the concept of combination prevention, in which condoms and other behavioural interventions are promoted alongside biomedical and seroadaptive advancements [59]. Specifically, future prevention work should build upon the array of prevention strategies actually being used by gay men and encourage strategic, as opposed to consistent, condom use [46]. Providing gay men with multiple strategies to optimize safety while also leaving space for subjectivity and agency in sexual decision-making would potentially improve intervention uptake.

The importance of emotional factors in sexual decision-making described by the participants in this study indicates that optimal HIV prevention work must also expand beyond knowledge promotion and cognition. Consistent with the existing literature [7, 23, 60], participants’ desires for intimacy, trust, and erotic pleasure – especially within the perceived safety of primary relationships – served as strong motivators for CAI, often taking priority over avoiding sexual risk. However, a large proportion of HIV transmissions among gay men occur within primary relationships, challenging the notion of such relationships as low risk [48, 49]. There is therefore a need for intervention work that addresses the emotional motivators of sexual risk and speaks to ongoing relationships as a potential HIV risk context [7, 26, 34, 47].

The emergence of community-developed seroadaptive strategies provides further opportunity for intervention work as gay men increasingly utilize these strategies in the absence of condoms [44]. The vast majority of our participants used seroadaptive strategies as a major part of their prevention regimen, despite their concerns that the effectiveness of these strategies relied on trust and honest communication. This reliance on accurate, honest disclosure can be problematic: many gay men fail to have explicit conversations about serostatus and viral load, relying instead on social cues and intuition with both casual and longer-term partners [12, 14, 19, 33, 61]. The effectiveness of seroadaptive strategies can be further complicated by factors such as substance use and peer pressure to engage in CAI or limit communication prior to sex [21, 44]. Interventions aimed at decreasing CAI should therefore teach gay men to improve their communication and sexual negotiation skills [17]; such skills could also increase the efficacy of seroadaptive strategies by minimizing incidences in which a partner’s serostatus is incorrectly assumed [61]. Intervention efforts should offer strategies to enable disclosure conversations between partners and assist gay men in advocating for their own sexual safety. Since seroadaptive strategies are already widely used and will presumably continue to be so, improving the efficacy of these strategies and promoting them within a broader combination prevention approach will likely improve prevention efforts as a whole.

With the decline in consistent condom use and the increasing prevalence of other prevention strategies, intervention efforts must also aim to clearly and succinctly convey the efficacy of these strategies [6–10, 62, 63]. In their review of seroadaptive practices, Cassels and Katz suggest that 14–44% of HIV positive gay men and 25–38% of HIV negative gay men already engage in serosorting alone [61]. Participants in this study utilized a variety of seroadaptive strategies ranging from established modes of prevention – like serosorting and seropositioning – to methods less supported by scientific evidence [46, 64] – such as withdrawal prior to ejaculation, urination after sex, and limiting the duration of sex while in the receptive role. Existing research suggests that seroadaptive strategies are more effective than indiscriminate CAI and less effective than consistent condom use, but additional work is needed [61–63]. With accurate data, health officials and community organizations would then be able to provide gay men with the information required to make educated risk evaluation decisions. Since seroadaptive strategies rely on accurate knowledge of one’s HIV status, education efforts should also continue to encourage regular HIV and STI testing [61, 63].

Finally, our analysis indicates that HAART-based advances may be shifting gay men’s perceptions of “risky” sexual partners. Study participants viewed sex with HIV positive undetectable partners as lower risk than sex with those who claimed to be HIV negative and argued that disclosure of HIV status was more trustworthy when it came from an undetectable partner. They also believed such partners to be more connected to healthcare and therefore more aware of their holistic sexual health. While this trend toward utilizing a partner’s viral load to inform sexual risk decisions has been observed in other studies [33, 44, 45, 61], the dominance of viral load sorting over serosorting among our participants seems unusually high compared to previous research [63]. It is likely attributable in large part to the promotion of TasP in BC [51, 65], which emphasizes that the use and acceptability of various prevention strategies is highly contextual.

As with all analyses, the strengths and limitations of this study must be considered. A qualitative examination of condom use habits among a small group of HAART-experienced participants in Vancouver, BC is not generalizable to other contexts or even to Vancouver’s gay population as a whole. However, the findings of this study provide a detailed picture of the complex and contradictory perceptions and practices influencing gay men’s condom use with nuances that quantitative models are not able to capture. While participants’ disclosure of condom use habits and CAI may have been influenced by social desirability bias that would likely have produced an overrepresentation of stated condom use, we endeavoured to minimize the potential impacts of this bias through the use of peer interviewers and a semi-structured interview technique [52, 53, 55]. Our team approach to thematic analysis and use of Lincoln and Guba’s trustworthiness criteria for evaluating qualitative research supports the validity of our analysis [54, 55].

## Conclusions

The findings of this study indicate that the expansion of prevention options available to gay men has begun to redefine traditional interpretations of sexual “risk” and “safety,” with perceptions and use of condoms influenced by multiple factors on all levels of the social ecological model of health. Condoms, once synonymous with safer sex, are no longer viewed as the sole effective means of HIV prevention; many gay men feel that combining biomedical and seroadaptive strategies is an effective alternative that allows for greater sexual freedom and pleasure. Simultaneously, the notion of “risky” HIV positive partners has shifted in light of viral load sorting and the emerging category of undetectability. In this context, public health and community-based interventions that advocate solely for strict condom adherence will likely be viewed as obsolete. Future research must address the full range of gay men’s prevention strategies, providing evidence on the efficacy of these individual strategies and their optimal use within a combination prevention approach. In turn, future public health and community-based intervention work will need to translate this information into prevention messages while assisting gay men in developing the communication and advocacy skills needed to optimize these emerging strategies.

## Additional file

Additional file 1: NPT Qualitative Questions HIV Negative. [HIV Negative] ARV-based Prevention Interview Guide. The interview guide used with HIV negative participants in the Momentum Study who had experience using ARV prevention strategies. (DOCX 27 kb)
