# Supplementary material for: “Condoms are … like public transit. It’s something you want everyone else to take”: Perceptions and use of condoms among HIV negative gay men in Vancouver, Canada in the era of biomedical and seroadaptive prevention
Source: BMC Public Health. 2019 Jan 28;19:120. doi: 10.1186/s12889-019-6452-7 (PMC6350294; doi:10.1186/s12889-019-6452-7)
Supplement: Supplementary file 1 — NPT Qualitative Questions HIV Negative. [HIV Negative] ARV-based Prevention Interview Guide. The interview guide used with HIV negative participants in the Momentum Study who had experience using ARV prevention strategies. (DOCX 27 kb) [file 12889_2019_6452_MOESM1_ESM.docx]

### [HIV Negative] ARV-based Prevention Interview Guide

**Objective**: To explore the perspectives of HIV-negative “early adopters” of ARV-based prevention strategies (nPEP, PrEP, “TasP-informed”) as they relate to knowledge development, decision-making to use, experiences with, and impacts of these strategies, particularly as they relate to substance use and sexual behaviours.

**Eligibility criteria:** Momentum participants who are currently using PreP, have previously used nPEP, and/or report only having condomless sex with other GBMSM on treatment or with low viral loads.

***nPEP***: “stands for Post Exposure Prophylaxis. It’s a treatment that may stop you from contracting HIV after risky sex”

***PrEP*** “stands for Pre-Exposure Prophylaxis. It’s a treatment that may reduce the chances of you contraction HIV if taken before risky sex”

***TasP***: treatment as prevention

***OPENING QUESTION OPTIONS***

1. ***What place does sex have in your life?***
2. ***What connection do you have to the gay community?***
3. ***Where did you learn about HIV?***
4. **What are some ways that you prevent getting HIV? That other gay men do to prevent HIV infection?**

*(prompts: have you heard of guys using ARVs to prevent infection?)*

**What does prevention mean to you?**

1. **How [where/from whom] did you learn about these prevention approach(es)?**

*(prompts: partners, friends, agencies, healthcare providers, social media, Internet, school, TV, etc.)*

**Have you used any of these approaches yourself? (PreP, PEP, TasP-informed)**

*(prompts: Why? Why not? What helped you make this decision? What didn’t help?)*

***[Repeat question block for each tool/strategy as needed]***

**Talking specifically about your use of: 1) PreP, 2) nPEP, and/or 3) Tasp/ undetectable/viral load sorting:**

1. **What experiences have you had using this approach?**

*(Positive prompts: pleasure, freedom, less anxiety)*

*(Negative prompts: side effects, cost, stigma, adherence, access issues, substance use)*

1. **What are/were the parts you liked about this prevention strategy? What were the best parts of this prevention strategy? What are/were the hardest/worst? How do they compare?**

*(prompts: Has using these approaches/tools impacted other areas of your health? does this have any relation with your substance use?)*

1. **What experiences, if any, have you had talking to partners/potential partners about this?**

*(prompts: How did they react? What questions or concerns did they have? How did you educate them? Why did you not talk to partners about them? How did your conversation affect a sense of trust?)*

1. **How has your idea of “risky” and “protected” sex changed now that TasP/PrEP/PEP exist?**

***[End of question block]***

***[If used viral load/TasP]***

1. **How do you get information about your sexual partner(s)’ viral load or whether they are on ARV treatment? (What info do you want or need?)**

*(prompts: Do you trust the information? How confident are you about what your sexual partner(s)’ tell you about their viral load or whether they are on ARV treatment? How do you verify the info is accurate?)*

1. **Have you had any partners that were on PrEP?**
2. **Do you use a combination of HIV prevention approaches?**

*(prompts: How do you decide which prevention measure to use with which partner? Are there times you use certain strategies and other times you use others? how is this affected by or related to substance use?)*

1. **Would you consider using any other prevention approaches?**

*(prompts: Which ones? Why those ones? Why not other ones? What would make you more likely to use them? Do you plan on using just one, or a combination? How do you compare between approaches, or decide which ones to use? What role do condoms have?)*

**[If not mentioned or explored yet…]**

1. **Have you ever tried to access nPEP and/or PrEP?**

*(prompts: Can you describe the process of getting access to nPEP and/or PrEP? Were you successful in getting the prescription? Where did you get it from?)*

1. **What barriers or challenges did you face?**

*(prompts: Who or what was helpful? Did your financial situation and/or health insurance coverage affect accessing nPEP and/or PrEP?)*

1. **What was it like to talk to a healthcare provider about nPEP and/or PrEP?**

*(prompt: How much did your doctor know about your sex life before talking about PrEP? Did you bring it up or did your doctor? Are you out to your doctor?)*

1. **What was the main thing that influenced your decision to use (or not use) nPEP and/or PrEP?**

*(prompts: what kind of community support did you have? What kind of community support do you want? Did you know other users? What was your relationship with agencies and case workers?)*

***[New question block]***

1. **How effective or ineffective do you feel different approaches are in terms of prevention (TasP vs. PrEP vs. PEP vs. condoms vs. sero-sorting etc.)?**

*(prompts: Why do you feel that way? What or who inspires confidence? What doesn’t? What would make you feel more confident about their effectiveness?)*

1. **Why aren’t other gay and bi men trying these approaches (TasP, PrEP and PEP)?**

*(prompts: Why or why not? How does this affect your use of these approaches? What are some reasons people may not find these approaches acceptable?)*

1. **What are the positive and negative implications of using these approaches (TasP, PrEP and PEP)?** *(prompt to talk about the opposite aspects)*

*(prompts: how do you think these might work for guys using substances?)*

1. **Do you talk to other people about these approaches (Tasp, PrEP and PEP)?**

*(prompts: Why not? Who do you talk to? How did you bring this topic up? How did those conversations go? What/who would make you feel more comfortable to talk to other people about this? Where would you feel comfortable talking about this?)*

1. **Have TasP/PrEP/PEP changed your substance use? sex life/sexual practices?**

*(prompts: The type of sex you would have, whether or not you would use condoms, the frequency of sex, the types of partners, and communication with partners and/or health care providers, whether you use substance when parting or having sex)*

1. **How has your idea of “risky” and “protected” sex changed now that TasP/PrEP/PEP exist? What role do condoms have in your like now?**
2. **Do you plan to use any of TasP/PrEP/PEP in the future?**

*(prompts: If no, why not? What would be the ideal way to access them? What would be the ideal way to use them? What kind of community support would you want?)*
